# Processing different types of iconicity in Chinese transferred epithet comprehension: An ERP study

**DOI:** 10.3389/fpsyg.2022.1032029

**Published:** 2022-12-22

**Authors:** Qiaoyun Liao, Mengting Gao, Xin Weng, Quan Hu

**Affiliations:** ^1^Institute of Linguistics, Shanghai International Studies University, Shanghai, China; ^2^School of English Studies, Sichuan International Studies University, Chongqing, China

**Keywords:** transferred epithet, comprehension, iconicity of markedness, ERPs, similarity feature

## Abstract

Transferred epithet can be regarded as a reflection of semantic markedness since the modifier and the modified conflict with each other and lead to semantic deviation; yet the corresponding processing mechanism is less studied. The present study examined the neurocognitive mechanism of Chinese transferred epithet comprehension by employing ERP technique from the perspective of Iconicity of Markedness. Participants were required to read materials with different types of semantic markedness, namely unmarked linguistic expression (literal sentences) and marked linguistic expression (transferred epithets), and then judge whether the targets were words or pseudo-words. In terms of semantic markedness, the targets are words reflecting the unmarked semantic meaning of literal sentences and marked semantic meaning of transferred epithets respectively. The target words after transferred epithets elicited a larger N400 and a smaller LPC than those in literal sentences. These results suggest that processing sentences with marked and unmarked iconicity involve different neural mechanisms, with the former requiring more cognitive efforts to extract the similarity features.

## 1. Introduction

Transferred epithet (*yijiu* in Chinese), one of frequently used rhetorical devices, is defined as “a figure of speech in which the epithet is transferred from the appropriate noun to modify another to which it does not really belong” (Cuddon, 1979, p.85). In other words, it involves the transference of a modifier of one thing to another. For example, in the expression of “*sleepless night*,” the adjective “*sleepless*,” which is used to describe animate beings, is transferred to modify the inanimate noun “*night*,” enabling it to possess the property of animate beings.

The transferring in descriptive modifiers, considered as a “betrayal” of the regular grammar in rhetoric (Wang, 2013), is a kind of illogical modification. It is considered as a variable of conceptual metaphors from the perspective of cognitive linguistics (Sun et al., 2017), and is interpreted as the outcome of the cross-space mapping involving the establishment of mental spaces of the modifier and modified (Wang, 2005; Wang, 2013) based on the Conceptual Integration Theory (Fauconnier and Turner, 1994). Since the illogical collocation is at odds with logical rules, the transference of modifiers can convey implicit meanings, which creates esthetic and pragmatic effects with context provided, such as effectively attracting a receiver’s attention, expressing human emotion, and arousing a rich imagination depending on the association of proximity (Nesfield and Wood, 1964, p. 271). Although these studies have explored transferred epithets from a theoretical perspective, little has been known about the processing mechanism of Chinese transferred epithets. In addition, few studies explored the processing of Chinese transferred epithets from the perspective of Iconicity of Markedness that provides a satisfactory explanation to this unconventional linguistic expression with semantic anomaly.

Iconicity emphasizes the resemblance relationship between the form of a linguistic sign and the object or idea it represents (Dingemanse et al., 2020), which is considered as the conceived similarity between conceptual structure and linguistic forms (Haiman, 1985, p. 1; Tabakowska, 1999, p. 410). Moreover, there are various principles of iconicity among which the “Iconicity of Markedness” targets at the linguistic expressions that are morphologically, syntactically or semantically marked. For example, Wang (1998) considered that iconicity based on markedness in linguistic expressions can be related to semantic markedness. Specifically, unmarked linguistic expressions convey conventional and general meaning, requiring less cognitive resources for comprehension; as for marked linguistic expressions, with distinctive features and complex structures, usually deviate from normal expression and convey extra and abnormal meaning, which is more complicated. In other words, for semantic markedness, unmarked semantic categories in language are reflected as those prototypical properties which are experienced and acquired first in our development (Langendonck, 2007, p. 401), whereas marked semantic ones are attributed to schemata-clashes occurring in our concepts at first sight, and then the possibility could be found among the impossibility, conveying certain esthetic effects.

Seen from the perspective of “Iconicity of Markedness” based on semantic markedness, transferred epithets can be regarded as marked linguistic expressions with semantic deviation from conventional meanings, as the literal meaning of the modifier and the modified conflict with each other, exhibiting odd syntagmatic relations due to the semantic tension of their immediate constituents (Sun et al., 2017). Consider the following examples in Chinese:

1. 一个即将倒闭的工厂 (*a bankrupting factory*)
2. 一个即将死亡的工厂 (*a dying factory*).

In daily use, example (1) is a linguistic expression that is in accordance with reality and meets our expectation, since “*bankrupting*” conveys exactly the conventional meaning of “*insolvency or unable to pay the debt*.” Thus it is an expression with unmarked iconicity, because “*bankrupting*” presents the highest semantic relatedness with its conventional meaning. Yet in example (2), the modifier “*dying*,” meaning “*to stop living or existing*,” usually modifies animate beings; yet it is used to modify “*factory*,” a non-animate object, which transfers a conventional collocation to a novel one and deviates from its conventional meaning. In this way, transferred epithets, such as “*dying factory*,” have less semantic relatedness with its conventional meaning, which can be regarded as expressions with marked iconicity in terms of its semantic markedness.

With such a semantic deviation and cognitive inconsistency within “*dying factory*,” how can transferred epithets be comprehended? According to Sun et al. (2017), transferred epithets, being variables of conceptual metaphors, possess all the essences of metaphors, and involve at least two different conceptual fields which feature special image schemata. Based on the idea that the core of transferred epithet is transferring properties from a vehicle to a topic, the semantic incongruity indicates the contrast of imageries from two conceptual fields. However, with metaphorical thinking and appropriate contexts, transferred epithets can be reconciled to a pragmatically harmonious and coherent interpretation. Given this map, we hold that transferred epithets may possess a similar processing approach to metaphors. When comprehending transferred epithets with the form of “Adjective + Noun” (e.g., “dying factory”), people may first retrieve the conventional meaning of “*dying*” for modifying animate beings (i.e., “to stop living or existing”) and then encounter semantic contradictions when integrating the adjective and noun in transferred epithets. In order to solve the semantic clash and continue processing, they are prone to search for the similarity feature, which is a critical and salient feature that people deliberately choose among the conceived similarities between the two conceptual fields involved in transferred epithets, constructing concrete and abstract similarity and likeness (Pietarinen, 2008) between them. For example, based on the two conceptual fields of “*a dying man*” and “*a dying factory*,” readers can extract the similarity feature of “*getting worse and worse*” by matching “*a dying man is a man whose health condition is getting worse and worse*” and “*a dying factory is a factory which is getting worse and worse*.” With this similarity feature, the association of these two conceptual fields is triggered, and a coherent interpretation such as “*a businessman’s factory is dying just like a man is dying*” can be constructed. In this way, with people’s subjective factors in work, including emotion and experience stored in their long-term memory, the semantic inconsistency pre-imposed by “*dying factory*” is resolved by the interpretation of “*dying*” in “*dying factory*” in terms of “*insolvency*.”

Based on the above analysis, we intend to consider that iconicity of markedness provides a new way to investigate the comprehension of transferred epithets by regarding them as marked linguistic expressions with semantic deviation. Yet studies on iconicity and transferred epithets provide inadequate evidence with regard to such a perspective. Specifically, studies on iconicity mainly focus on vocal and sound iconicity (Lockwood et al., 2016; Perlman and Lupyan, 2018; Vigliocco et al., 2019; Johansson et al., 2021), iconicity of lexicon and signed language (Wilcox, 2004; Thompson et al., 2009; Ortega, 2017; Pretato et al., 2018; Dingemanse et al., 2020), syntax (Marcus and Calude, 2010; Levshina, 2015), and affective iconicity (Ullrich et al., 2016; Aryani et al., 2018, 2019). Also, previous ERP studies on iconicity mainly reported iconicity-related N400. For example, a smaller N400 is elicited for matched gestures compared to mismatched gestures with speech (Habets et al., 2011; Drijvers and Özyüre, 2018), and for onomatopoeic words in comparison to the non-onomatopoeic words (Peeters, 2016), reflecting facilitated access for matched gestures and onomatopoeic words with iconic mappings between form and meanings. In addition, only two studies employing ERP techniques explore the iconic representation of metaphors from the perspective of semantic anomaly (Balconi and Tutino, 2007; Balconi and Amenta, 2010). In Balconi and Tutino (2007), literal and metaphor meanings (e.g., “The lawyers are professional/sharks”) were compared. Results showed that there was no significant difference between literal and metaphor meaning in N400; however, N300 was sensitive to metaphorical meaning with a more negative deflection than literal meaning, indicating that metaphor possessed imagery-based iconicity. Balconi and Amenta (2010) further explored the frozen metaphor by comparing literal (e.g., “A fighter is a soldier”) with metaphoric expressions (e.g., “A fighter is a lion”). They observed two negative deflections (the N300-N400 complex), but only N300 showed an enhanced negativity for metaphoric decoding. It again suggested that metaphors were more iconic and more imagery-based than literal ones as reflected by the N300 index.

As mentioned above, we hold that transferred epithets may have similar construal approach to metaphors. Previous ERP studies on metaphor processing have mainly reported three ERP components: N300, N400, and LPC. Firstly, N300 detected in metaphor processing is related to imagery as discussed above. Secondly, N400 is a centro-parietal negativity typically observed between 300 and 500 ms after the onset of a stimulus and reflects semantic violation or anomaly (Kutas and Hillyard, 1980). In metaphor processing, a larger N400 is elicited by metaphors as compared to literal expressions, which is interpreted as a representation of semantic conflicts between source domains and target domains, resulting in an increased processing difficulty (Pynte et al., 1996; Coulson and Van Petten, 2002; Lai et al., 2009; Goldstein et al., 2012). Thirdly, LPC is a positive-going wave emerging around 500–900 ms after stimulus onset and reflects processes engaged in meaning reanalysis (Friederici et al., 1999). This metaphor-related LPC as compared with literal expressions is related to the extra efforts required to integrate meanings in a figurative context (Kazmerski et al., 2003; Rutter et al., 2012; Bambini et al., 2019; Jankowiak et al., 2021). Taken together, this study mainly focuses on the N300, N400, and LPC components.

In sum, based on the Iconicity of Markedness, the present study aimed to investigate whether and how linguistic expressions with different types of semantic markedness (i.e., transferred epithets vs. literal expressions) are processed by using ERP techniques. Specifically, we used a target word to represent the final integrated meaning of the critical sentences, which manifests unmarked and marked semantic meanings of the adjectives in the “Adjective + Noun” of literal expressions and transferred epithets, respectively. In other words, this target word conveys the conventional meaning of the adjectives in literal sentences and the deviated meaning of the adjectives in transferred epithets, forming unmarked and marked semantic iconicity, respectively. In this way, the processing of the target word represents the processing of different types of semantic markedness. Also, we hypothesized that when processing the target word, participants may first detect a semantic incongruity between the meaning of the target word and the meaning of the adjective in transferred epithets. Then by referring to the context, the similarity features between the two conceptual fields involved in the transferred epithets can be extracted, with which the previous semantic incongruity can be reconciled and the coherent meaning of transferred epithets can be obtained. During this process, N300, N400, and LPC were used as signals to detect the potential differences between the processing of the target words in the above two conditions.

## 2. Materials and methods

### 2.1. Participants

A total of 20 postgraduate students (12 females, mean age = 24 years, 22–26 years) from Sichuan International Studies University, Chongqing, China participated in this experiment and received financial rewards. All were native Chinese speakers and were completely unaware of the purpose of the present experiment. All were right-handed and had normal or corrected-to-normal vision, and had no speech-hearing, neurological or psychiatric disorders. Written informed consent was obtained from all participants prior to participation. Two subjects’ data were excluded due to excessive artifacts.

### 2.2. Materials

Since prototypical Chinese transferred epithets are presented in the “Adjective + Noun” expression (Li, 2010, p.35), the present study uses “Adjective + Noun” as the form to convey key information of literal sentences and transferred epithets. A total of 40 sets of materials were created (see the sample of materials in Table 1), each containing two sentence conditions (i.e., transferred epithets and literal sentences). Each sentence consisted of a context, a critical sentence, a target word, and a statement. The context (e.g., “Xiao Pan is forced to do housework at home, so every day after school,”) served as an introduction sentence that described a specific situation or event. The following critical sentence contained the “Adjective + Noun” form that was either a literal expression (e.g., “He goes home with a slow pace.”) or a transferred epithet (e.g., “He goes home with a lazy pace.”). After the critical sentence, as mentioned before, a target word (e.g., “delay”) was created to represent the final integrated meaning of the critical sentences, showing different semantic meanings of the critical sentences, especially the “Adjective” in the “Adjective + Noun” form. Specifically, as for the adjective in the literal sentences (e.g., “slow”), it is closely related and highly similar to the target word (e.g., “delay”), because “delay” conveys the unmarked conventional meaning of “slow”; however, as for the adjective in transferred epithets (e.g., “lazy”), it is not closely related or similar to the target word, because “delay” conveys the marked unconventional meaning of “lazy.” Thus in this way, the use of target word allowed us to detect the processing of different types of semantic markedness and the extraction of similarity features within the same linguistic item. Finally, a statement (e.g., “Xiao Pan does not like doing housework.”), which was about the content of the previous scenario, was added to detect whether participants have concentrated on the experiment and understood the materials or not.

**Table 1:** Experimental stimuli sample.

Condition	Example stimuli			
	Context	Critical sentence	Target	Statement
Transferred epithet sentence	小潘在家里就会被迫做家务, 所以每天放学后,	他就迈着懒惰的步伐回家.	拖延	小潘不喜欢做家务.
	*Xiao Pan zaijiali jiuhui beipo zuojiawu*, *souyi meitian fangxuehou*,	*ta jiu maizhe landuode bufa huijia.*	*tuoyan*	*Xiao Pan bu xihuan zuojiawu.*
	Xiao Pan is forced to do housework at home, so every day after school,	he goes home with a lazy pace.	delay	Xiao Pan does not like doing housework.
Literal sentence	小潘在家里就会被迫做家务, 所以每天放学后,	他就迈着缓慢的步伐回家.	拖延	小潘不喜欢做家务.
	*Xiao Pan zaijiali jiuhui beipo zuojiawu, souyi meitian fangxuehou*,	*ta jiu maizhe *huanmande bufa* huijia.*	*tuoyan*	*Xiao Pan bu xihuan zuojiawu.*
	Xiao Pan is forced to do housework at home, so every day after school,	he goes home with a slow pace.	delay	Xiao Pan does not like doing housework.

Manipulated words are underlined for expository purposes.

Based on the 40 sets of materials, two validation tests were conducted. Firstly, a total of 20 postgraduate students from Sichuan International Studies University who did not participate in the ERP experiment were invited to rate the acceptability of the materials on a 5-point scale from 1 (the least unacceptable) to 5 (the most acceptable). They were instructed to rate the acceptability on the basis of whether the sentences were semantically coherent and pragmatically appropriate. Transferred epithet sentences and literal sentences with mean scores less than 4 were excluded in order to ensure that the materials were highly acceptable. Secondly, as for the target word, another 20 graduate students from Sichuan International Studies University who did not participate in the ERP experiment and the first test were invited to rate the semantic relatedness between the targets and the adjectives of “Adjective + Noun” form in the critical sentences on a 5-point scale from 1 (highly unrelated) to 5 (highly related). An average score between 2.3 and 3.7 for transferred epithet sentences and between 3.7 and 5.0 for literal sentences were chosen as experimental stimuli so as to ensure that the target word can reflect the unmarked conventional meaning of the literal sentences and the marked deviated meaning of transferred epithets.

At last, 21 sets of materials were selected as final experimental materials. The mean scores of sentence acceptability for the transferred epithet sentences and literal sentences were 4.5 (*SD* = 0.09) and 4.6 (*SD* = 0.14) respectively, with no significant difference [*t*(19) = 2.008, *p* > 0.05]. The mean scores of semantic relatedness between the targets and the adjectives in transferred epithets and literal sentences were 3.4 (*SD* = 0.19) and 4.4 (*SD* = 0.15) respectively, with a significant difference [*t*(19) = −15.289, *p* < 0.001]. Meanwhile, the number of Chinese characters in the context sentences varied from 16 to 21, and critical sentences from 9 to 15. The targets consisted of two Chinese characters, and the statements varied from 7 to 14 Chinese characters.

Besides, 14 sentences were added as fillers, which had the same structure as those formal experimental materials except that their target words were pseudo-words. For example, for this filler sentence “Xiao Hu’s wife is giving birth in the hospital, and he is waiting for her. The anxious waiting makes him in terrible fidgets.,” the target word could be “*列初* (*lie chu*).” Moreover, all materials were repeated once in order to obtain enhanced experimental effects. Thus the entire study constituted in 42 sets of experimental materials and 28 filler sentences, leading to 112 sentence stimuli in general. For each participant, these 112 stimuli were pseudo-randomized in a way that sentences of the same condition would not appear three times consecutively.

### 2.3. Procedure

Participants were tested individually in a dimly lit and quiet room while seated in a comfortable chair. For each trail, a fixation cross “+” lasting 500 ms was displayed to remind participants that the experiment was about to begin. Then the context sentence was presented for 3,500 ms and followed by a blank of 500 ms. Afterwards, the critical sentence was presented for 2,000 ms followed by a blank of 500 ms. Next, the target was presented until the participants pressed a button to decide whether it was a word or a pseudo-word (“F” for word and “J” for pseudo-word). If the participants did not respond within 500 ms, the following blank screen with a question mark remained until they made a response. The reason that this task was adopted was because the words or pseudo-words judgment was made based on the semantic level of the targets (Lin et al., 2002) and could keep the targets the same in two conditions. Then the final statement was presented for the participants to judge whether the statement was in accordance with previous context (“F” for True and “J” for False) after a 500 ms blank. “F” and “J” buttons were counterbalanced across subjects with half subjects pressing “F” for true, the other half pressing “F” for false. A new trial started after a 600–800 ms blank. The stimuli were presented in four blocks with each containing 28 trials, and short breaks were allowed between blocks. The participants were provided with six practice trails to be familiar with the experiment.

### 2.4. EEG recording and data analysis

The electroencephalogram (EEG) was continuously recorded (band pass 0.05–100 Hz, sampling rate 1,000 Hz) from 64 Ag/AgCl electrodes held in place on the scalp by an elastic cap with a ground electrode on the medial frontal line and references on the left and right mastoid (Luck, 2014, p. 154). Vertical and horizontal electro-oculograms were recorded. Electrode impedance was maintained below 5 kΩ throughout the experiment.

EEG signals were sampled at 500 Hz and filtered with a bandpass of 0.1–30 Hz (24 dB/octave). The EEGs were re-referenced offline to the average of all electrodes (Luck, 2014). The EEG was segmented in epochs of 1,200 ms, time-locked to target (i.e., the final probe word following the critical sentence, see Table 1) onset and included a 200 ms pre-stimulus baseline. Trials contaminated by amplifier clipping, bursts of EMG activity, or peak-to-peak deflection exceeding ±100 μV were excluded from averaging.

**Figure 1:**
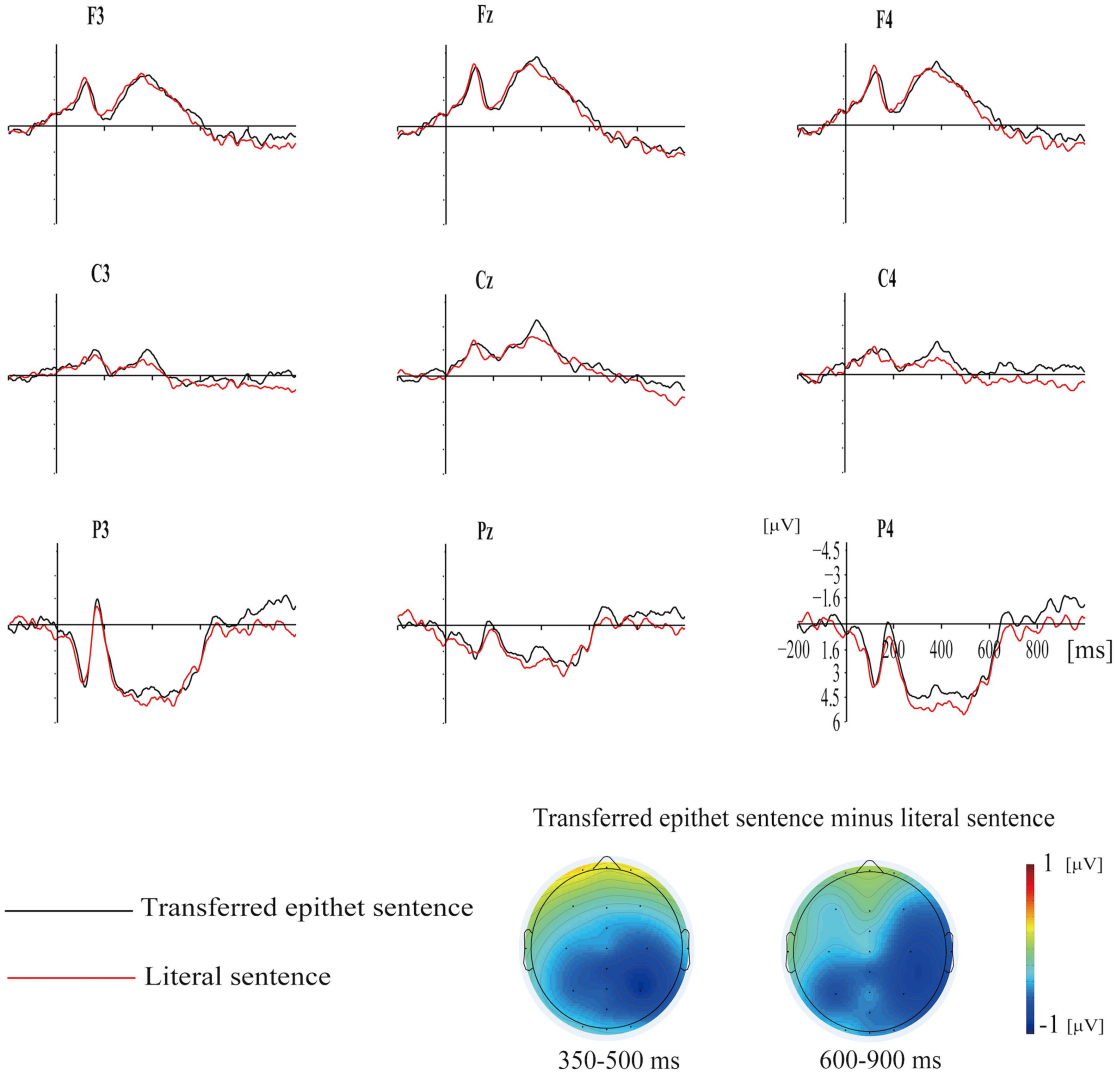
Grand average ERPs of the target words in two conditions with topographic maps for difference waves (transferred epithet sentence minus literal sentence) in the 350–500   ms and 600–900                     ms time windows.

Nine representative electrodes were chosen for data analysis based on previous ERP studies on iconicity (Vigliocco et al., 2019) and rhetorics such as metaphor (Tang et al., 2017a,b) and irony (Spotorno et al., 2013; Caillies et al., 2019). According to the overall averages (see Figure 1) and previous studies, the analyses of two time windows including N400 (350–500 ms) and LPC (600–900 ms) were conducted, as no N300-related waves were detected. A repeated measures ANOVA with the sentence type (transferred epithet sentences; literal sentences), hemisphere (left: F3, C3, P3; midline: Fz, Cz, Pz; right: F4, C4, P4), and region (anterior: F3, Fz, F4; central: C3, Cz, C4; posterior: P3, Pz, P4) as repeated factors was conducted on the mean amplitudes of these two time windows. Greenhouse–Geisser corrections were applied to the degrees of freedom where necessary.

## 3. Results

### 3.1. Behavioral data

The mean reaction times (RTs) and accuracy of judgment for each condition were presented in Table 2. Analyses showed that there was no significant difference between the mean RTs of targets for the transferred epithet condition and literal condition [*t*(17) = −0.870, *p* > 0.39], and the accuracy of targets between them was not significant [*t*(17) = 1.458, *p* > 0.16]. Meanwhile, the accuracy of statement judgment between them revealed no significant difference [*t*(17) = 1.000, *p* > 0.33], suggesting that participants concentrated on the experiment and understood the materials.

**Table 2:** Means and standard deviations of the average RT and accuracy.

Condition	Target words		Statement
	Mean RTs (ms)	Accuracy(%)	Accuracy(%)
Transferred epithet sentence	907.639 ± 175.902	100.0 ± 0.000	98.7 ± 0.019
Literal sentence	937.487 ± 161.403	99.7 ± 0.008	97.9 ± 0.030

### 3.2. Electrophysiological data

#### 3.2.1. 350–500 ms

Analysis of ERP data in this time window yielded a significant main effect of sentence type [*F*(1, 17) = 6.142, *p* < 0.03, *η_p_^2^* = 0.265], and the amplitude of N400 elicited by the transferred epithet condition (*M* = −0.610, *SD* = 1.945) was larger than that of the literal condition [*M* = 0.591, *SD* = 1.777; *t*(17) = −5.263, *p* < 0.001]. No other significant interactions were found (*ps* > 0.09; see Figure 1).

#### 3.2.2. 600–900 ms

Analysis showed a significant main effect of sentence type [*F*(1, 17) = 4.710, *p* < 0.05, *η_p_*^2^ = 0.217], and a significant interaction of sentence type × hemisphere [*F*(2, 34) = 4.345, *p* < 0.03, *η_p_*^2^ = 0.204]. Follow-up simple effects ANOVAs revealed that the transferred epithet condition evoked smaller late positive amplitudes than the literal condition at right sites [*F*(1, 17) = 11.187, *p* < 0.005, *η_p_*^2^ = 0.397; see Figure 1].

## 4. Discussion

Based on the Iconicity of Markedness, the current study aimed to explore the neurocognitive mechanism of Chinese transferred epithet comprehension by employing ERP technique. Results revealed significant differences in the N400 and LPC amplitudes elicited by the targets following transferred epithets (i.e., marked linguistic expressions) and literal sentences (i.e., unmarked linguistic expressions). These results suggest that processing sentences with different types of iconicity involve different neural mechanisms.

### 4.1. Different neural mechanisms of processing sentences with marked and unmarked iconicity

At the behavioral level, it was found that the targets following transferred epithets were processed as quickly as those following literal sentences. As for this null effect, we intend to suggest that it might be related to the high familiarity of transferred epithets used in the current study. In this experiment, conventional transferred epithets are selected, which means that they are fairly familiar to participants. Thus participants are able to process them in a rapid way just as they process literal sentences. Therefore, with a high familiarity of transferred epithets, longer response time is not necessarily required as compared with literal sentences. However, similar response times are not equal to similar neural cognitive responses; thus further ERP signals are recorded and analyzed.

At the neural-cognitive level, the kernel issue examined here is the larger N400 and smaller LPC elicited by the targets following transferred epithets than those following literal sentences. A larger N400 in the transferred epithet condition is in line with the studies that report enhanced N400 effects in metaphor processing (Pynte et al., 1996; Coulson and Van Petten, 2002; Lai et al., 2009; Wang, 2009), contributing to the idea that more cognitive operations are required to resolve the semantic incongruity of figurative sentences. To some extent, this is also similar to the results of iconicity processing in both signed language (Habets et al., 2011; Drijvers and Özyüre, 2018) and spoken language (Peeters, 2016), with a smaller N400 reflecting the access of iconic mappings between forms/sounds and meanings. In the current experiment, the enhanced N400 may reflect the semantic incongruity detected by participants when they tried to integrate the targets into the previous contexts of transferred epithets, as the meaning of targets reflect the marked deviated meaning of the adjectives in the transferred epithets, triggering semantic inconsistency signaled by an increased N400.

Also, a decreased LPC in the condition of transferred epithets was also found in the current study. It is consistent with previous studies of metaphor processing (Kazmerski et al., 2003; Arzouan et al., 2007a,b; Rutter et al., 2012; Bambini et al., 2019). This decreased LPC is related to the processing of sentences with semantic anomalies that require secondary semantic integration processes (Friederici et al., 1999) and the processing of semantic integration of conceptually challenging materials that require a reinterpretation of the meaning after an initial failure during its comprehension (Goldstein et al., 2012; Tang et al., 2017a,b). In the current study, the decreased LPC for transferred epithets indicates that though participants encounter a semantic incongruity between targets and the transferred epithets at the initial stage as signaled by the previous N400 effect, the final integrated meaning expressed by the targets facilitates the ongoing processing after the similarity features are extracted (see section 4.2), providing an access to resolve the previous semantic incongruity.

Furthermore, the current N400 and LPC effects which represent the different neural mechanisms for the processing of marked and unmarked semantic iconicity are different from the results of the studies of metaphorical iconicity with an enhanced N300 effect (Balconi and Tutino, 2007; Balconi and Amenta, 2010). One possible explanation for the absence of N300 in the present study can be ascribed to the fact that unlike metaphors, transferred epithets do not have the constraints of imagery similarity that metaphor requires (Xu, 2011). That is to say, metaphor is thought to have many image-like characteristics (Pietarinen, 2008); thus the comprehension of metaphor is mainly based on the similarity between the images of the topic and the vehicle. However, the similarity involved in transferred epithets is more abstract, because the choice of the “Adjective” is decided by the speaker’s psychological states and feelings caused by the perceptual quality of things or objects represented by the “Noun” in a certain context. In another word, both metaphors and transferred epithets are concerned with iconicity, but the iconicity is more specific in metaphors than in transferred epithets. Given this map and considering that N300 is an image-specific component which represents the processing of pictures by the activation of an image-based system (Federmeier and Kutas, 2002; Hamm et al., 2002) and reflects the process of transposition from imagistic format into linguistic representation (West and Holcomb, 2002), it is reasonable that there was no N300 effect elicited in the current transferred epithet condition. Such a distinction indicates that the processing of transferred epithets does not totally resemble that of metaphors in that transferred epithets involve semantic inconsistency, further indicating the uniqueness of transferred epithets.

### 4.2. The underlying operation of processing transferred epithets: Similarity feature extraction

Iconicity represents a relation of similarity which itself is graded, that is, iconicity is a continuum going from less similar to more similar, with many degrees of iconicity between them (Waugh, 1994). Based on such an assumption, we argue that during the above discussed process of processing Chinese transferred epithets, what underlies this cognitive operation is the suitable similarity considerations (Pietarinen, 2008).

In the present study, the consideration of similarity is achieved by the extraction of certain similarity features that match the interpretations of the two conceptual fields involved in transferred epithets. Specifically, as for literal sentences, the targets represent the exact conventional meaning of the adjectives in the “Adjective + Noun” of the literal condition, which directly shows the meanings expressed in literal context. As a result, participants encounter no semantic inconsistency when they try to integrate the targets into the literal contexts.

However, as for the transferred epithet condition, the targets could not be easily integrated with the previous context as the target word represents deviated and abnormal meanings of the adjectives of the transferred epithets. When such an incongruity is detected, the extraction of similarity features is activated. Firstly, in the detection of the semantic incongruity between targets and transferred epithets, participants are able to recognize the two conceptual fields involved in transferred epithets (Sun et al., 2017), such as “*a lazy man*” and “*a lazy pace*” involved in the transferred epithet condition of Table 1. Secondly, based on the two related conceptual fields and the overall contexts, participants search the key similarity feature of these two fields with the consideration of similarity (Pietarinen, 2008). For example, “*lazy*” can convey the disapproving meaning such as “not willing to work or make any efforts” and the approving meaning such as “easy and relaxed” at the same time. From these semantic meanings, participants refer to the contexts and then choose the feature of “*unwillingness*” that could match the common properties of “*a lazy man not willing to work or making any efforts*” and “*a lazy pace showing Xiao Pan’s unwillingness to do housework*.” Thirdly, based on such a matched similarity feature, the semantic incongruity occurring at the first stage can be resolved as participants integrate “*lazy*” into “*pace*” by constructing a coherent interpretation such as “*a lazy pace relates to the unwillingness to do something just as a man is lazy*.” Thus further processing of the target “*delay*,” whose meaning is “postpone doing something due to unwillingness,” can be carried out as participants are able to obtain the meaning of “*lazy*” in terms of the final integrated meaning conveyed by the target word “delay.”

In this way, after the detection of semantic inconsistency, participants are able to continue the processing of the target words following transferred epithets by searching and matching the similarity features of the two conceptual fields in certain contexts, promoting emergent properties to reconcile the previous semantic incongruity and then achieve coherent interpretations. Therefore, based on the current results and discussions, we propose that the key operation underlying the processing of Chinese transferred epithets is the extraction of similarity features.

## 5. Conclusion

The present study explored the neurocognitive mechanism of Chinese transferred epithet processing based on Iconicity of Markedness. The differences signaled by N400 and LPC in transferred epithet condition as compared with literal condition suggest that processing sentences with different types of semantic markedness involve different neural mechanisms, with the former requiring more cognitive efforts to extract the similarity features. It provides electrophysiological evidence for Chinese transferred epithet comprehension.

## Data availability statement

The original contributions presented in the study are included in the article/supplementary material, further inquiries can be directed to the corresponding authors.

## Ethics statement

The studies involving human participants were reviewed and approved by Institute of Linguistics, Shanghai International Studies University. The patients/participants provided their written informed consent to participate in this study.

## Author contributions

QL, MG, XW, and QH contributed to the study conception and design, wrote the paper, and commented on previous versions of the manuscript. Material preparation, data collection, and analysis were performed by QH. All authors contributed to the article and approved the submitted version.

## Funding

This work was supported by the Key Program of the National Social Science Fund of China (grant no. 19AYY011), the Major Scientific Program of Shanghai International Studies University (grant no. 2018114027), the 5th Tutor Guidance Program of Shanghai International Studies University (grant no. 2022113002), and the Postgraduate Research Projects of Sichuan International Studies University (grant no. SISU2018YZ03).

## Conflict of interest

The authors declare that the research was conducted in the absence of any commercial or financial relationships that could be construed as a potential conflict of interest.

## Publisher’s note

All claims expressed in this article are solely those of the authors and do not necessarily represent those of their affiliated organizations, or those of the publisher, the editors and the reviewers. Any product that may be evaluated in this article, or claim that may be made by its manufacturer, is not guaranteed or endorsed by the publisher.
